# Investigating alpha‐synuclein co‐pathology in Alzheimer's disease by means of cerebrospinal fluid alpha‐synuclein seed amplification assay

**DOI:** 10.1002/alz.13658

**Published:** 2024-02-07

**Authors:** Giovanni Bellomo, Andrea Toja, Federico Paolini Paoletti, Yihua Ma, Carly M. Farris, Lorenzo Gaetani, Nicola Salvadori, Davide Chiasserini, Anna Lidia Wojdaƚa, Luis Concha‐Marambio, Lucilla Parnetti

**Affiliations:** ^1^ Center for Memory Disturbances Lab of Clinical Neurochemistry Section of Neurology Department of Medicine and Surgery University of Perugia Perugia Italy; ^2^ R&D Unit, Amprion Inc San Diego California USA; ^3^ Section of Physiology and Biochemistry Department of Medicine and Surgery University of Perugia Perugia Italy

**Keywords:** Alzheimer's disease, biomarkers, cerebrospinal fluid, neuropsychological evaluation, seed amplification assay, synucleinopathy

## Abstract

**INTRODUCTION:**

Lewy body disease, a frequently observed co‐pathology in Alzheimer's disease (AD), can be identified *antemortem* in cerebrospinal fluid (CSF) by α‐synuclein seed amplification assay (αS‐SAA). The prevalence and clinical impact of CSF αS‐SAA positivity in AD are still unknown.

**METHODS:**

αS‐SAA was performed on CSF samples from 240 AD patients (preclinical, prodromal, and dementia stages), 85 controls, 84 patients with Parkinson's disease (PD), and 21 patients with PD with dementia or dementia with Lewy bodies. In AD patients, associations between αS‐SAA positivity and cognitive changes were also evaluated.

**RESULTS:**

In agreement with available neuropathological studies, αS‐SAA positivity was observed in 30% of AD patients (vs 9% in controls), and was associated with cognitive decline, visuospatial impairment, and behavioral disturbances.

**DISCUSSION:**

αS‐SAA positivity in AD patients reflects the prevalence observed in neuropathological series and is associated with a worse clinical outcome. These data confirm the validity of CSF αS‐SAA positivity as biomarker of synucleinopathy.

## 1 INTRODUCTION

Alzheimer's disease (AD) and Parkinson's disease (PD) are the most common neurodegenerative proteinopathies of the central nervous system.^1^ Lewy body (LB) disease (LBD) is the neuropathological hallmark of clinically defined diseases like PD, PD with dementia (PDD), and dementia with Lewy bodies (DLB). LBD includes LBs and neurites that are mainly composed of aggregated misfolded α‐synuclein (αSyn).^2^ A recent neuropathology study of 1153 AD brains showed that nearly 33% of subjects with AD had concomitant LBD.^3^ This co‐pathology has been retrospectively associated to parkinsonian manifestations and to a more aggressive form of AD, sometimes called AD Lewy body variant.^4^ Cerebrospinal fluid (CSF) soluble misfolded αSyn aggregates (αSyn‐seeds) are a well‐accepted biomarker for synucleinopathies like PD and DLB.^5^, ^6^ αSyn‐seeds can be reliably detected in CSF by an in vitro technique called αSyn Seed Amplification Assay (αS‐SAA),^7^, ^8^, ^9^ which enables the unprecedented possibility of identifying underlying αSyn pathology in living patients and evaluating if co‐pathology modulates clinical presentation. In particular, αS‐SAA has shown high sensitivity and specificity in detecting LB pathology from *antemortem* CSF in neuropathology studies.^10^, ^11^ The identification of individuals with AD who also have LB co‐pathology has become increasingly critical, especially given the availability of disease‐modifying treatments. Understanding this frequent co‐pathology is imperative as we strive to address adverse events and treatment ineffectiveness in certain patients undergoing anti‐amyloid drugs, which remain poorly understood.

In this study, we analyzed a large AD cohort including preclinical stage (pre‐AD) patients, those with mild cognitive impairment due to AD (MCI‐AD), and AD dementia (AD‐dem) patients. Control (CTRL) subjects as well as patients with PD, PDD, and DLB were included as well. Our aim was to estimate the prevalence of CSF αS‐SAA positivity in AD patients and its influence on clinical parameters and outcome.

## 2 METHODS

### 2.1 Study population

All subjects included in this study were enrolled at the Neurology Clinic of the University of Perugia (Italy). The composition of the cohort is reported in Table 1. All patients underwent medical history, physical and neurological examination, brain imaging (computed tomography or magnetic resonance imaging), and lumbar puncture for the measurement of CSF core AD biomarkers. All patients underwent a thorough neuropsychological evaluation including the Mini‐Mental State Examination (MMSE)^12^ and the Italian version of the HIV dementia scale (HDS‐IT)^13^ as screening tests, plus a comprehensive neuropsychological battery for the assessment of executive functions (Frontal Assessment Battery, FAB),^14^ attention and working memory (Trail Making Test: TMT‐A, TMT‐B^15^; digit span forward and backward^16^), learning and memory (Rey Auditory Verbal Learning Test [RAVLT],^17^ short story recall of Anna Pesenti^18^), language (phonemic fluency^17^ and category fluency^18^), and visuo‐constructional abilities (copying of drawings with and without landmarks from the Mental Deterioration Battery^17^; Clock Drawing Test [CDT]^19^). The Neuropsychiatric Inventory (NPI)^20^ and Clinical Dementia Rating scale^21^ were also administered to assess behavioral changes and clinical staging, respectively. CSF core AD biomarkers, namely the β‐amyloid (Aβ)1‐42/Aβ1‐40 ratio (Aβ42/40 ratio), phosphorylated tau protein at threonine 181 (p‐tau181), and total tau (t‐tau), were measured in all patients.

**RESEARCH IN CONTEXT**

1. **Systematic review**: The literature has been reviewed using conventional sources (eg, PubMed and Google Scholar). The literature suggests that Lewy bodies are neuropathologically present in a high percentage of Alzheimer's disease (AD) patients, and that they can modulate AD clinical course.
2. **Interpretation**: We found a significant proportion of AD patients (30%) being positive to α‐synuclein seed amplification assays (αS‐SAAs). Moreover, we found a significant association between assay positivity and visuospatial impairment. We found αS‐SAA positivity present in all clinicals stages of AD (even in the preclinical phase, 27% positivity rate). Notably, αS‐SAA positivity was associated with a more marked cognitive decline at follow‐up.
3. **Future directions**: Stratifying AD patients based on CSF αS‐SAA may help in selecting more homogeneous cohorts of patients for clinical studies and detect AD patients who may also benefit from therapies targeting synucleinopathy.

**TABLE 1:** Demographic features, MMSE scores, and αS‐SAA outcome in the clinical groups considered.

Group	Age (years)	M/F	MMSE	αS‐SAA outcome			Total
				Pos.	Neg.	Inc.	
CTRL	68.5 ± 8.8	45/40	26.7 ± 2.7	8 (9%)	74 (87%)	3 (4%)	**85**
CTRL‐CN	65.3 ± 9.7	26/17	28.2 ± 1.2	2 (5%)	38 (88%)	3 (7%)	**43**
CTRL‐MCI	71.8 ± 6.5	19/23	25.4 ± 3	6 (14%)	36 (86%)	0 (0%)	**42**
AD	72.6 ± 6	79/161	21 ± 6	72 (30%)	160 (67%)	8 (3%)	**240**
pre‐AD	73 ± 5.8	7/15	28.2 ± 1.1	6 (27%)	16 (73%)	0 (0%)	**22**
MCI‐AD	72.3 ± 5.3	40/81	23.5 ± 3.1	31 (26%)	85 (70%)	5 (4%)	**121**
AD‐dem	72.8 ± 6.7	32/65	15.9 ± 5.7	35 (36%)	59 (61%)	3 (3%)	**97**
typical AD	73 ± 5.7	65/140	21.7 ± 5.5	60 (29%)	139 (68%)	6 (3%)	**205**
lv‐PPA	71.7 ± 6.5	8/14	14.3 ± 6.8	3 (14%)	18 (82%)	1 (5%)	**22**
PCA‐AD	67.4 ± 7.9	5/7	19.2 ± 6.8	8 (67%)	3 (25%)	1 (8%)	**12**
fv‐AD	69.0	1/0	21.0	1 (100%)	0 (0%)	0 (0%)	**1**
PD/DLB	67 ± 7.1	71/34	26 ± 4	91 (87%)	13 (12%)	1 (1%)	**105**
PD‐CN	64.8 ± 6.5	22/18	28.3 ± 1.6	36 (90%)	4 (10%)	0 (0%)	**40**
PD‐MCI	66.9 ± 6	29/15	26.4 ± 1.8	38 (86%)	6 (14%)	0 (0%)	**44**
PDD/DLB	71.7 ± 8.4	20/1	20.4 ± 5.6	17 (81%)	3 (14%)	1 (5%)	**21**

*Note*: Age and MMSE are represented as mean ± standard deviation; αS‐SAA positive, negative, and inconclusive outcomes are reported both as numbers and as percentages (in parentheses). AD subgroups comprise one set based on clinical stage (pre‐AD, MCI‐AD, AD‐dem) and another based on clinical presentation (typical AD, lv‐PPA, PCA‐AD, fv‐AD) for the same group of 240 patients.

Abbreviations: αS‐SAA, α‐synuclein Seed Amplification Assay; AD, Alzheimer's disease; AD‐dem, Alzheimer's disease at dementia phase; CTRL, controls; CTRL‐CN, controls, cognitively normal; CTRL‐MCI, controls, with mild cognitive impairment, stable after 2‐years, with normal CSF profile and other neurodegenerative disorders excluded; DLB, dementia with Lewy bodies; fv‐AD, frontal variant AD clinical presentation; Inc., inconclusive αS‐SAA; lv‐PPA, logopenic variant of primary progressive aphasia AD presentation; MCI‐AD, mild cognitive impairment due to AD; MMSE, Mini‐Mental State Examination; Neg., negative αS‐SAA; PCA‐AD, posterior cortical atrophy AD clinical presentation; PD‐CN, cognitively normal Parkinson's disease; PDD, Parkinson's disease with dementia; PD‐MCI, mild cognitive impairment associated to Parkinson's disease; Pos., positive αS‐SAA; pre‐AD, preclinical Alzheimer's disease.

The AD cohort consisted of 240 patients. AD was diagnosed according to the CSF biomarker profile A+/T+, independent of the clinical stage, in line with the 2018 National Institute of Aging–Alzheimer's Association criteria.^22^ Combining the CSF profile (A+/T+) with neuropsychological evaluation and functional assessment, AD patients were grouped as (1) pre‐AD (*n* = 22); (2) prodromal, that is, MCI‐AD (*n* = 121); and (3) AD‐dem (*n* = 97).^22^ According to the clinical presentation, *n* = 205 of these same patients had the typical amnestic form (typical AD), *n* = 22 had the logopenic variant of primary progressive aphasia (lv‐PPA),^23^ *n* = 12 had posterior cortical atrophy (PCA‐AD),^24^ and *n* = 1 had the frontal variant (fv‐AD).^25^

PD/PDD/DLB patients (n = 105) were diagnosed according to the current diagnostic criteria.^26^, ^27^, ^28^ Based on neuropsychological assessment, PD patients were categorized as cognitively normal PD (PD‐CN) or PD with MCI (PD‐MCI).^29^

The CTRL patients (n = 85) underwent lumbar puncture for CSF analysis within the diagnostic work‐up (headache, mononeuropathy, psychiatric disturbances, subjective cognitive complaints). Their CSF profile was negative for AD (A−/T−). According to the neuropsychological evaluation, these subjects were either cognitively unimpaired (CTRL‐CN) or had MCI (CTRL‐MCI). Only CTRL‐MCI subjects with stable cognitive performances at 2‐year follow‐up and a negative ^18^F‐fluorodeoxyglucose‐positron emission tomography result were included.

#### 2.1.1 Consent statement

All the procedures involving human subjects were performed following the Declaration of Helsinki. All participants gave written informed consent to use medical data and biomaterials for research purposes. The study was approved by the local Ethics Committees (Comitato Etico Aziende Sanitarie Regione Umbria 19369/AV and 20942/21/OV).

### 2.2 CSF collection, A/T/N biomarkers analysis, and total protein concentration

Lumbar puncture was performed according to international guidelines^30^; 10 to 12 mL of CSF was collected in sterile polypropylene tubes (Sarstedt tubes, code: 62.610.210) and centrifuged for 10 minutes (2000 × g), at room temperature. Aliquots of 0.5 mL were frozen at −80◦C in polypropylene tubes (Sarstedt tubes, code: 72.730.007). CSF samples were analyzed on the fully automated chemiluminescent platform Lumipulse G600‐II (Fujirebio Inc) for Aβ42, Aβ40, t‐tau, and p‐tau181 levels in the Laboratory of Clinical Neurochemistry of the University of Perugia. All CSF samples were analyzed directly in their 0.5 mL storage tubes. The Aβ42/40 ratio was used as the CSF biomarker for amyloidosis since it represents a more robust marker compared to Aβ42 alone.^31^ We used the cutoffs previously calculated in our Center.^32^ Total protein concentration was evaluated by means of Pierce 660 nm Protein Assay Reagent cat. 22,660 (Thermo Scientific, USA).

### 2.3 αS‐SAA

The αS‐SAA developed by Concha‐Marambio and colleagues was performed at Amprion Inc. The αS‐SAA was performed as previously reported,^5^, ^33^, ^34^ with some modifications. CSF samples were evaluated in triplicate (40 μL/well) in a clear bottom 96‐well plate containing a reaction mix consisting of 0.3 mg/mL recombinant α‐Syn (Amprion, cat# S2020), 100 mM PIPES pH 6.50 (Sigma, cat# 80,635), 500 mM NaCl (Lonza, cat# 51,202), 10 μM ThT (Sigma, cat# T3516), and two 1/8‐inch Si_3_N_4_ beads (Tsubaki Nakashima). The assay was performed in a BMG FLUOstar Omega shaker/reader at 42°C, with 15‐minute shaking/incubation cycles. Maximum fluorescence (Fmax) from three replicates was used for result determination; if all three replicates present Fmax higher than 3,000RFU, the sample is deemed positive. If only two cross the 3,000RFU threshold, the sample is considered inconclusive. If one or no replicate presents Fmax higher than 3,000RFU, the sample is considered negative. For αS‐SAA‐positive AD patients, the following SAA‐derived parameters (averaged on three replicates) were included in kinetic analysis: Fmax, time‐to‐threshold (TTT, time to reach 3000 RFU), F24h (fluorescence at 24 h), AUFC (area under the fluorescence curve), Smax (maximum slope of the fluorescence curve), TSmax (time to reach the maximum slope), and AUFCdydx (area under the derivative of the fluorescence curve). Parameters were estimated using the Omega data analysis tool Mars (BMG).

### 2.4 Statistical analysis

After excluding subjects with inconclusive αS‐SAA outcomes (*n* = 12 in total), Fisher's exact test for count data and logistic regression (LR; assuming age and sex as covariates) were used to assess the significance of αS‐SAA positivity prevalence among clinical groups. Since these two tests always provided consistent response in terms of *p*‐values, we simply reported those referring to LR, which were also adjusted for age and sex (adj. *p*). LR (assuming age and sex as covariates) was used also to assess the significance of the association between neuropsychological scores, neuropsychiatric symptoms, and levels of core biomarkers of AD with αS‐SAA positivity. The same approach was applied to determine whether cognitive worsening was associated with αS‐SAA positivity. Spearman's correlation and partial correlation (CSF total protein concentration as covariate) analyses were applied to assess the association between αS‐SAA kinetic parameters, neuropsychological scores, and neuropsychiatric symptoms. False discovery rate adjustments were not performed directly on *p*‐values in order not to apply an overly stringent penalty to the results of this explorative study, which considered a very high number of clinical and experimental variables. However, the effects of applying Benjamini‐Hochberg correction (BHC)^35^ in each of the analyses performed are discussed in the Results section. During the BHC process, we took into account the close definition and the substantial correlation observed among AUFC, AUFCdydx, Smax, F24h, and Fmax (*ρ* values of ∼0.9) in AD patients with positive αS‐SAA outcomes. Similarly, we considered the strong correlation between TTT and TSmax. In light of these high correlations, we treated these kinetic parameters as two independent variables for testing purposes. Meanwhile, all the clinical scores under consideration were treated as independent variables. The statistical analyses were conducted using R version 4.3.1 and OriginPro version 9.

## 3 RESULTS

**FIGURE 1 alz13658-fig-0001:**
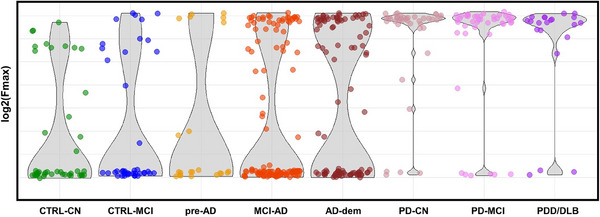
Overview of αS‐SAA maximum fluorescence (Fmax) in all clinical groups considered. Violin/scatter plots displaying log2 of Fmax values averaged on three replicates. αS‐SAA, α‐synuclein seed amplification assay; AD‐dem, Alzheimer's disease at dementia phase; CTRL‐CN, controls, cognitively normal; CTRL‐MCI, controls, with mild cognitive impairment, stable after 2‐years, with normal CSF profile and other neurodegenerative disorders excluded; DLB, dementia with Lewy bodies; MCI‐AD, mild cognitive impairment due to AD; PD‐CN, cognitively normal Parkinson's disease; PDD, Parkinson's disease with dementia; PD‐MCI, mild cognitive impairment associated to Parkinson's disease; pre‐AD, preclinical Alzheimer's disease.

**FIGURE 2 alz13658-fig-0002:**
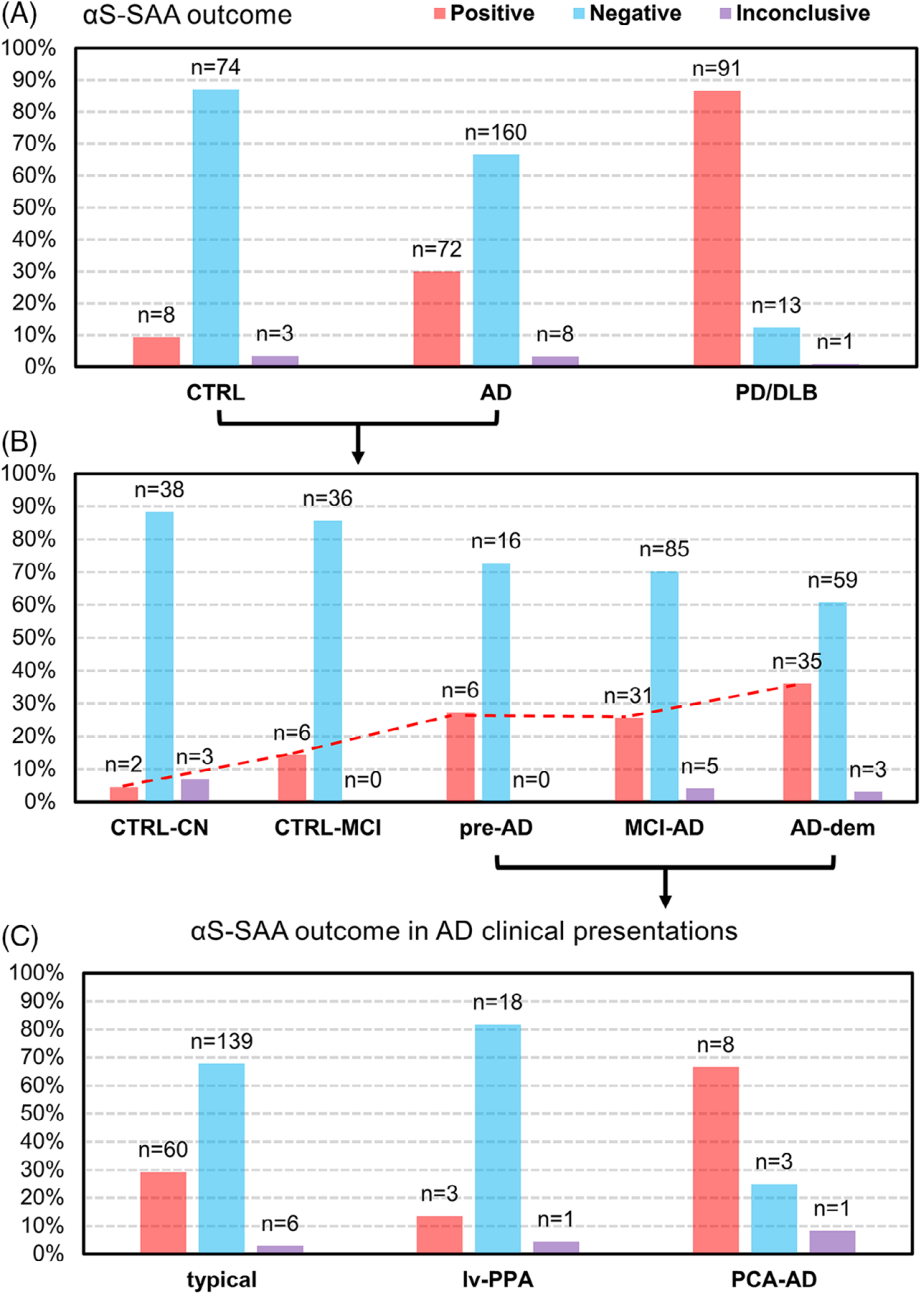
CSF αS‐SAA outcome in the clinical groups and subgroups considered. (A) Bar plot representing the percentages of CSF αS‐SAA positive, inconclusive, and negative subjects for CTRL, AD, and PD/DLB groups. (B) Bar plot representing the percentages of CSF αS‐SAA positive, inconclusive, and negative subjects for CTRL‐CN, CTRL‐MCI, pre‐AD, MCI‐AD, and AD‐dem subgroups. (C) Bar plot representing the percentages of CSF αS‐SAA positive, inconclusive, and negative subjects among typical/amnestic AD, lv‐PAA, and PCA‐AD clinical variants. The absolute number of positive, negative and inconclusive outcomes is reported on the top of each bar. αS‐SAA, α‐synuclein seed amplification assay; AD, Alzheimer's disease; AD‐dem, Alzheimer's disease at dementia phase; CSF, cerebrospinal fluid; CTRL, controls; CTRL‐CN, controls, cognitively normal; CTRL‐MCI, controls, with mild cognitive impairment, stable after 2‐years, with normal CSF profile and other neurodegenerative disorders excluded; lv‐PPA, logopenic variant of primary progressive aphasia AD presentation; MCI‐AD, mild cognitive impairment due to AD; PD, Parkinson's disease; PCA‐AD, posterior cortical atrophy AD clinical presentation; pre‐AD, preclinical Alzheimer's disease.

Basic demographic features, MMSE values, and αS‐SAA results for all the clinical groups are summarized in Table 1. αS‐SAA results were qualitatively compared among clinical groups in terms of Fmax averaged on the three replicates to graphically summarize the aggregation kinetics in αS‐SAA (Figure 1). In agreement with the available literature, the great majority of the PD, PDD, and DLB subjects (85% to 90%) presented high Fmax, consistent with αSyn‐seed detection. Subjects in the AD continuum presented both high and low Fmax, suggesting detection of αSyn‐seeds in some of the subjects. Interestingly, the proportion of subjects with middle and high Fmax increases with AD progression. Subsequently, clinical groups were compared based on the actual result of αS‐SAA (Figure 2A, Table 2). In agreement with the clinical diagnosis, 87% of the synucleinopathy group and 9% of the control group were positive. In the whole AD group, an αS‐SAA positivity prevalence of 30% was found, significantly higher than in the CTRL group (9%, adj. *p* = 0.0015). When dividing into subgroups, CTRL‐CN subjects presented the lowest αS‐SAA positivity (5%), which is comparable to positivity rates reported for healthy control subjects.^5^, ^36^ The positivity rate increased along with the progression of the AD continuum, up to 36% observed in the AD‐dem group. However, even by removing the AD‐dem subgroup, the positivity[Fig alz13658-fig-0001] prevalence in the AD versus the CTRL groups remained significant (adj. *p* = 0.005).

**TABLE 2 alz13658-tbl-0002:** Pairwise comparisons of positive/negative outcomes among groups and relative *p*‐values.

Comparison	*p*‐value fisher	adj. *p*‐value LR
PD/DLB versus CTRL	< < 0.00001	< < 0.00001
PD/DLB versus AD	< < 0.00001	< < 0.00001
AD versus CTRL	0.00010	0.0015
pre‐AD+MCI‐AD versus AD‐dem	0.11	0.089
pre‐AD+MCI‐AD versus CTRL	0.0030	0.0050
typical/amnestic versus lv‐PPA	0.14	0.12
typical/amnestic versus PCA‐AD	0.0059	0.013
lv‐PPA versus PCA‐AD	0.0018	0.0037

*Note*: *p*‐values were determined both by Fisher exact test for numerical data and by logistic regression (LR) by assuming age and sex as covariates (adj. *p*‐value).

Abbreviations: AD, Alzheimer's disease; AD‐dem, Alzheimer's disease at dementia phase; CTRL, controls; DLB, dementia with Lewy bodies; lv‐PPA, logopenic variant of primary progressive aphasia AD presentation; MCI‐AD, mild cognitive impairment due to AD; PCA‐AD, posterior cortical atrophy AD clinical presentation; PD, Parkinson's disease; pre‐AD, preclinical Alzheimer's disease; typical/amnestic, typical/amnestic AD clinical presentation.

Interestingly, CTRL‐MCI subjects showed higher αS‐SAA positivity compared to CTRL‐CN (14% vs 5%). All the above‐reported *p*‐values remained statistically significant after BHC.

In AD patients, αS‐SAA positivity was significantly associated with a poorer performance in the copying of drawings test, a test assessing visuospatial and constructional abilities, at baseline (adj. *p* = 0.0058). We also identified an association between αS‐SAA positivity and changes in the total NPI score (adj. *p* = 0.042). However, when accounting for multiple testing effects, this last association did not remain statistically significant. No relevant associations were found with CSF Aβ42/40, or p‐tau181, or t‐tau. αS‐SAA positivity was not associated with sex (*p* = 0.90), age (*p* = 0.67), and baseline MMSE score (adj. *p* = 0.097) in AD patients.

With respect to AD clinical presentation, patients with the PCA‐AD variant presented a significantly higher αS‐SAA positivity (67%) compared to the typical AD (29%) (adj. *p* = 0.013) and the lv‐PPA presentations (3/22, 13.7%, adj. *p* = 0.0037, Figure 2C). Our cohort included only 1 patient with fv‐AD, who was αS‐SAA positive. All the *p*‐values reported for this analysis remained statistically significant after BHC.

### 3.1 Kinetic analysis and baseline clinical parameters

In AD patients with[Fig alz13658-fig-0002], [Table alz13658-tbl-0002] positive αS‐SAA (*n* = 72), we tested the association between αS‐SAA kinetic parameters (ie, TTT, Fmax, F24h, AUFC, Smax, TSmax, AUFCdydx) and baseline neuropsychological scores (ie, MMSE, HDS‐IT, CDT, FAB, TMT‐A, TMT‐B, digit span forward and backward, RAVLT, phonemic and category fluency, copying of drawings with and without landmarks) by means of Spearman's correlation coefficients (see supplementary material, Table S1). The correlations with the highest magnitude among those calculated were those between HDS‐IT and AUFCdydx (*ρ* = −0.27; *p* = 0.035), and those between digit‐span backward and TTT (*ρ* = −0.35; *p* = 0.019) and TSmax (*ρ* = −0.35; *p* = 0.018). We also tested the association between αS‐SAA kinetic parameters and baseline neuropsychiatric symptoms (ie, NPI items and sum of the NPI items). Among these, the strongest were TTT with hallucinations (*ρ* = −0.26; *p* = 0.034), delusions (*ρ* = −0.31; *p* = 0.011), and nighttime behavior disturbances (*ρ* = −0.28; *p* = 0.021). Similar associations were found for TSmax (*ρ* = −0.27; *p* = 0.026, *ρ* = −0.30; *p* = 0.013, *ρ* = −0.27; *p* = 0.025, respectively). In a previous work we showed that CSF lipoproteins affect the kinetics of αSyn‐seed amplification in a concentration‐dependent manner, confounding potential correlations between seeding activity and clinical presentation.^37^ We identified total CSF protein concentration as a surrogate measurement for lipoproteins that could be used to control for lipoprotein confounding effects. Hence, we added CSF total protein concentration as a covariate and computed partial correlations (see supplementary material, Table S2). Most of the associations remained unaltered after adjusting for CSF total protein concentration, that is, between AUFCdydx and HDS‐IT, and between digit span backward and TTT and TSmax. Remarkably, associations between hallucinations, delusions, and nighttime behavior disturbances with TTT (*ρ* = −0.28; *p* = 0.021, *ρ* = −0.34; *p* = 0.006, *ρ* = −0.31; *p* = 0.010, respectively) and TSmax (*ρ* = −0.30; *p* = 0.014, *ρ* = −0.33; *p* = 0.007, *ρ* = −0.31; *p* = 0.011, respectively) were even slightly stronger after considering CSF total protein concentration as a covariate (for correlation coefficients see Table S2).

Adding CSF total protein concentration in the model resulted in additional weak associations with *p*‐values smaller than 0.05: hallucinations and nighttime behavior disturbances with AUFC (*ρ* = 0.25; *p* = 0.037 and *ρ* = 0.27; *p* = 0.027, respectively), and irritability with Fmax (*ρ* = 0.26; *p* = 0.032) and Smax (*ρ* = 0.28; *p* = 0.021). However, also due to the substantial array of neuropsychological scores considered, all the associations determined in kinetic analysis, apart from those between CSF total protein concentration and SAA kinetic parameters, did not remain statistically significant after applying BHC for multiple testing.

### 3.2 Follow‐up

**FIGURE 3:**
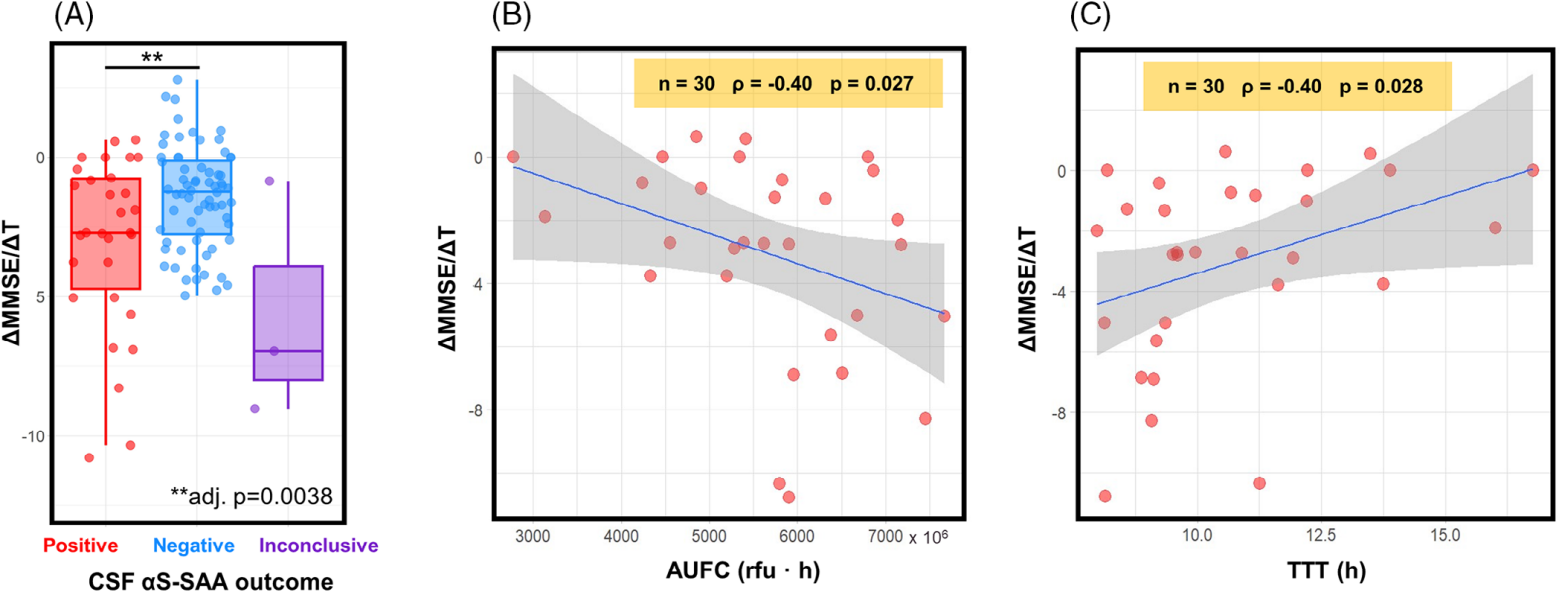
CSF αS‐SAA outcome and cognitive worsening in AD patients. (A) Box plots representing the rate of change of MMSE score over time (∆MMSE/∆t) in CSF αS‐SAA positive, negative, and inconclusive AD patients. Boxes represent the interquartile range, the horizontal lines within boxes represent the medians, and whiskers reflect the first/third quartile ± 1.5 times the interquartile range. The adj. *p*‐value reported is calculated by linear regression for pairwise comparisons adjusted for age and sex differences between groups. (B,C) Linear regression between ΔMMSE/ΔT and AUFC (B) and TTT (C) with 95% confidence intervals of the linear regression and Spearman's correlation coefficients (*ρ*) displayed. αS‐SAA, α‐synuclein seed amplification assay; AD, Alzheimer's disease; AUFC, area under the fluorescence curve; CSF, cerebrospinal fluid; MMSE, Mini‐Mental State Examination; TTT, time‐to‐threshold.

Among AD patients (*n* = 240), *n* = 106 underwent neuropsychological follow‐up. Specifically, *n* = 101 had available MMSE scores at least 2 years after the baseline MMSE (mean follow‐up = 2.9 years, standard deviation = 1.2 years), and *n* = 99 had available NPI scores at least 2 years after the baseline NPI (mean follow‐up = 2.6 years, standard deviation = 1.4 years). We evaluated if αS‐SAA positivity was associated with a greater rate of change of MMSE score over time (∆MMSE/∆t) and/or with a greater rate of change of global NPI over time using LR (Figure 3). No relevant associations were found between rate of change of NPI global score over time and αS‐SAA positivity or αS‐SAA kinetic parameters. There was a significant association between αS‐SAA positivity and MMSE worsening over time (adj. *p* = 0.0038). By considering just αS‐SAA‐positive patients, we found associations between ∆MMSE/∆t and TTT (*ρ* = −0.40; *p* = 0.028), TSmax (*ρ* = −0.40; *p* = 0.027, not shown in Figure 3, almost identical to TTT), and AUFC (*ρ* = −0.40; *p* = 0.027). Consistent with the previous findings, these associations were unaltered or became slightly stronger by accounting for CSF total protein concentration: TTT *ρ* = −0.41; *p* = 0.027, TSmax *ρ* = −0.40; *p* = 0.027, AUFC *ρ* = −0.45; *p* = 0.013. The *p*‐values reported for analyses regarding ∆MMSE/∆t remained statistically significant after BHC.

## 4 DISCUSSION

In this study, we used CSF αS‐SAA positivity to assess the prevalence of underlying synucleinopathy in a large and deeply characterized AD cohort, including all stages of the AD continuum (pre‐AD, MCI, and dementia) and atypical variants. αSyn‐seeds, indicative of an ongoing synuclein pathology,^11^ were detected in almost one out of three A+/T+ subjects.^22^ This prevalence is remarkably similar to the 33% of AD subjects with concomitant LBD reported by a large neuropathological analysis carried out in 1153 AD cases.^3^ These results, in addition to recently published data on a smaller AD cohort,^38^ demonstrate that a significant proportion of AD subjects have underlying αSyn pathology and this condition can be identified by αS‐SAA. Identification of αSyn pathology in vivo might help with better patient stratification and enrollment for clinical trials, as well as evaluating if αSyn pathology may influence the clinical response/outcome. To demonstrate the robustness of the applied assay, we also evaluated CSF samples from clinically diagnosed synucleinopathies (PD, PDD, and DLB) and cognitively normal controls from the same cohort. In agreement with previous studies, most PD/PDD/DLB patients were αS‐SAA positive and cognitively normal controls were negative^5^, ^36^, ^39^ for the vast majority, confirming the accurate performance of the αS‐SAA used in this study.

We also show that CSF αSyn‐seeds can be detected in preclinical AD subjects and at levels comparable to fully symptomatic AD subjects, suggesting that LB co‐pathology occurs in a significant proportion of subjects at any stage of AD. Moreover, we observed an increase in αS‐SAA positivity with disease progression, being highest in the dementia stage. Interestingly, control subjects with MCI presentedαS‐SAA positivity intermediate between pre‐AD and cognitively normal subjects. A longer follow‐up of these subjects will be critical to determine potential relations between αS‐SAA positivity in CTRL‐MCI subjects with phenoconversion to disease, disease progression, and/or disease outcome.

AD cases with positive αS‐SAA showed higher impairment of visuospatial skills at baseline and increased cognitive decline at follow‐up compared to αS‐SAA‐negative AD cases. Indeed, a marked reduction in MMSE scores was observed in αS‐SAA‐positive subjects despite the short follow‐up (2.9 years on average), which is consistent with a recent study demonstrating a faster worsening of global cognition in AD patients with concurrent LB pathology.^40^ Likewise, we found a much higher αS‐SAA positivity in subjects with the PCA‐AD variant (67%) than the typical (29%) and Iv‐PPA (14%) variants, which is consistent with the impairment in visual skills observed in the overall AD cohort. Analysis of additional subjects with atypical presentation is needed to corroborate this result, since we only analyzed 35 subjects without typical presentation. αS‐SAA positivity was also found to be weakly associated (unadjusted *p*‐value = 0.04) with altered NPI global score at baseline, which is consistent with retrospective studies linking NPI scores with LB co‐pathology in AD patients.^41^ These results suggest that αSyn pathology is not a bystander in AD, but an active part of the disease that influences clinical presentation.

The active role of underlying αSyn pathology in AD is also observed when evaluating associations between clinical presentation and αSyn seeding activity. Associations between kinetic parameters extracted from the αS‐SAA fluorescence curves and clinical parameters from the positive AD subjects were evaluated with and without CSF total protein as a covariate ^37^. Behavioral disturbances at baseline (hallucinations, delusions, nighttime behavior disturbances), were found to be associated with faster amplification (shorter TTT and TSmax).

Regarding follow‐up data, we observed a greater decline in MMSE scores among AD subjects who tested positive for αS‐SAA. This decline was particularly pronounced in those with faster αSyn‐seed amplification. In the absence of confounders, TTT or TSmax should be inversely proportional to the mass of αSyn seeds,^37^, ^42^, ^43^, ^44^ but individual CSF composition, that is, CSF lipoproteins or total protein concentration as a surrogate measure, has been shown to modulate αSyn aggregation kinetics in SAA.^6^, ^37^, ^43^ The associations described here became stronger in magnitude when including total CSF protein concentration as a covariate. To summarize the general outcome of our αS‐SAA kinetic analysis, a faster amplification of αSyn‐seeds was associated with greater worsening in clinical presentation of AD subjects.

The main strengths of our study are the large cohort of patients encompassing the entire AD clinical spectrum, from preclinical to dementia, and the deep characterization of the cohort including classical core AD biomarkers and neuropsychological assessment. Our study has limitations, which include the relatively short follow‐up of AD subjects, the lack of motor and smell scores for AD subjects, and the low proportions of αS‐SAA‐positive AD subjects with clinical follow‐up and AD subjects with atypical presentations. In addition, it should be considered that our exploratory study analyzed numerous variables, and it is important to note that *p*‐values resulting from kinetic analysis at baseline cannot be considered statistically significant when considering multiple testing effects. Therefore, further independent evaluations in larger cohorts are needed to validate our findings.

In conclusion, our study demonstrates that αSyn‐seeds can be detected in AD patients at all stages of the disease, including subjects considered prodromal and even preclinical. Importantly, AD subjects with an underlying αSyn pathology show worse clinical outcomes than those without co‐pathology. Also, some of these features seemed to associate with αSyn seeding activity measured by αS‐SAA kinetic parameters. Our data reinforce the evidence for the suitability of CSF αS‐SAA as a biomarker test for synucleinopathy,^6^ supporting the inclusion of SAA as a biomarker to demonstrate in vivo the presence of αSyn co‐pathology in AD patients. Accordingly, CSF αS‐SAA positivity is the most reliable candidate as a biomarker of synucleinopathy, to be added to the A/T/(N) system.

## CONFLICT OF INTEREST STATEMENT

The authors declare the following competing financial interests: Prof. Parnetti served as an Advisory Board Member for Fujirebio, IBL, Roche, and Merck. Dr. Concha, Ms. Farris, and Mr. Ma are inventors of several patents related to SAA technology (PMCA) and are associated with Amprion Inc., a biotech company focused on the commercial utilization of SAA for diagnosis. All other authors declare no financial and non‐financial competing interests. Author disclosures are available in the supporting information.

## Supporting information

Supporting Information

Supporting Information

## Data Availability

The data that support the findings of this study are available on request from the corresponding authors.
