# Supplementary material for: Investigating alpha‐synuclein co‐pathology in Alzheimer's disease by means of cerebrospinal fluid alpha‐synuclein seed amplification assay
Source: Alzheimers Dement. 2024 Feb 7;20(4):2444–52. doi: 10.1002/alz.13658 (PMC11032521; doi:10.1002/alz.13658)
Supplement: Supplementary file 1 — Supporting Information [file ALZ-20-2444-s001.docx]

***Supplementary material***

|  | **αS-SAA kinetic parameters** | | | | | | | | | | | | | |
| --- | --- | --- | --- | --- | --- | --- | --- | --- | --- | --- | --- | --- | --- | --- |
| **Neuropsychological  tests** | **TTT** | | **Fmax** | | **F24h** | | **AUFC** | | **Smax** | | **TSmax** | | **AUFCdydx** | |
|  | ρ | p-value | ρ | p-value | ρ | p-value | ρ | p-value | ρ | p-value | ρ | p-value | ρ | p-value |
| MMSE | -0.10 | 0.394 | -0.02 | 0.882 | -0.03 | 0.782 | 0.01 | 0.921 | -0.03 | 0.832 | -0.10 | 0.416 | -0.05 | 0.685 |
| HDS-IT | -0.11 | 0.388 | -0.20 | 0.114 | -0.23 | 0.072 | -0.10 | 0.447 | -0.19 | 0.148 | -0.11 | 0.388 | **-0.27** | **0.035** |
| CDT | 0.00 | 0.996 | -0.04 | 0.735 | 0.02 | 0.858 | 0.04 | 0.751 | -0.08 | 0.539 | 0.00 | 0.998 | 0.04 | 0.733 |
| TMT-A | 0.10 | 0.546 | -0.14 | 0.404 | -0.14 | 0.393 | -0.09 | 0.587 | -0.17 | 0.320 | 0.10 | 0.566 | -0.12 | 0.475 |
| TMT-B | -0.09 | 0.604 | -0.21 | 0.230 | -0.27 | 0.123 | 0.02 | 0.887 | -0.25 | 0.142 | -0.08 | 0.639 | -0.24 | 0.168 |
| DigitspanFORW | -0.15 | 0.316 | 0.10 | 0.522 | -0.01 | 0.929 | 0.14 | 0.370 | 0.13 | 0.401 | -0.15 | 0.318 | -0.03 | 0.827 |
| DigitspanBACK | **-0.35** | **0.019** | -0.07 | 0.656 | -0.21 | 0.178 | 0.13 | 0.392 | -0.04 | 0.782 | **-0.35** | **0.018** | -0.23 | 0.137 |
| RAVLT imm | 0.00 | 0.982 | 0.12 | 0.430 | 0.08 | 0.610 | 0.05 | 0.762 | 0.13 | 0.388 | 0.00 | 0.980 | 0.07 | 0.622 |
| RAVLT del | -0.01 | 0.924 | 0.07 | 0.617 | 0.15 | 0.318 | 0.05 | 0.742 | 0.08 | 0.605 | -0.01 | 0.923 | 0.16 | 0.295 |
| RAVLT-TrueRecog | -0.11 | 0.492 | 0.25 | 0.095 | 0.25 | 0.102 | 0.23 | 0.134 | 0.27 | 0.076 | -0.11 | 0.459 | 0.25 | 0.095 |
| RAVLT-FalseRecog | -0.07 | 0.668 | 0.09 | 0.556 | 0.01 | 0.960 | 0.16 | 0.283 | 0.07 | 0.657 | -0.05 | 0.768 | 0.04 | 0.813 |
| SR | -0.05 | 0.807 | 0.01 | 0.950 | 0.08 | 0.679 | 0.01 | 0.965 | -0.01 | 0.960 | -0.04 | 0.815 | 0.07 | 0.714 |
| Draw copy | -0.11 | 0.472 | 0.11 | 0.477 | -0.04 | 0.800 | 0.10 | 0.508 | 0.10 | 0.494 | -0.10 | 0.491 | -0.03 | 0.823 |
| Draw copy land | -0.13 | 0.392 | 0.03 | 0.837 | -0.02 | 0.919 | 0.07 | 0.616 | 0.07 | 0.622 | -0.12 | 0.398 | -0.02 | 0.911 |
| Letter fluency | 0.01 | 0.937 | -0.05 | 0.747 | -0.02 | 0.868 | -0.08 | 0.598 | 0.00 | 0.978 | 0.01 | 0.926 | -0.04 | 0.784 |
| Cat fluency | -0.13 | 0.391 | 0.09 | 0.539 | 0.03 | 0.858 | 0.09 | 0.560 | 0.08 | 0.595 | -0.12 | 0.399 | 0.03 | 0.839 |
| CDR | 0.12 | 0.344 | 0.13 | 0.318 | 0.18 | 0.170 | 0.04 | 0.753 | 0.11 | 0.416 | 0.12 | 0.363 | 0.22 | 0.090 |
| **NPI domains** |  |  |  |  |  |  |  |  |  |  |  |  |  |  |
| Anxiety | -0.05 | 0.689 | 0.04 | 0.717 | -0.03 | 0.789 | 0.10 | 0.404 | 0.06 | 0.652 | -0.04 | 0.746 | -0.03 | 0.830 |
| Delusions | **-0.31** | **0.010** | -0.14 | 0.266 | -0.19 | 0.120 | 0.12 | 0.316 | -0.13 | 0.298 | **-0.30** | **0.013** | -0.18 | 0.136 |
| Hallucinations | **-0.26** | **0.034** | -0.04 | 0.719 | -0.07 | 0.553 | 0.18 | 0.153 | -0.03 | 0.807 | **-0.27** | **0.026** | -0.08 | 0.522 |
| Depression/dysphoria | 0.13 | 0.274 | 0.00 | 0.971 | 0.03 | 0.788 | -0.11 | 0.371 | 0.01 | 0.967 | 0.14 | 0.240 | 0.03 | 0.827 |
| Agitation/aggression | -0.02 | 0.879 | -0.19 | 0.123 | -0.17 | 0.167 | -0.14 | 0.258 | -0.17 | 0.158 | -0.01 | 0.938 | -0.15 | 0.218 |
| Euphoria | -0.20 | 0.095 | -0.15 | 0.208 | -0.20 | 0.101 | 0.00 | 0.973 | -0.15 | 0.236 | -0.20 | 0.095 | -0.20 | 0.094 |
| Apathy/indifference | 0.04 | 0.727 | -0.08 | 0.530 | -0.03 | 0.817 | -0.05 | 0.683 | -0.05 | 0.696 | 0.04 | 0.734 | -0.01 | 0.923 |
| Disinhibition | -0.13 | 0.278 | 0.14 | 0.265 | 0.03 | 0.826 | 0.20 | 0.099 | 0.13 | 0.301 | -0.13 | 0.280 | 0.05 | 0.673 |
| Irritability | 0.14 | 0.266 | 0.09 | 0.483 | 0.18 | 0.152 | -0.08 | 0.532 | 0.09 | 0.474 | 0.13 | 0.287 | 0.16 | 0.189 |
| Aberrant motor behavior | -0.03 | 0.816 | -0.03 | 0.793 | -0.10 | 0.421 | -0.04 | 0.766 | -0.04 | 0.734 | -0.03 | 0.787 | -0.08 | 0.503 |
| Nighttime behaviors | **-0.28** | **0.021** | -0.04 | 0.737 | -0.05 | 0.673 | 0.17 | 0.158 | -0.04 | 0.762 | **-0.27** | **0.025** | -0.04 | 0.756 |
| Appetite/eating | 0.11 | 0.351 | 0.00 | 0.973 | -0.01 | 0.926 | -0.05 | 0.662 | -0.02 | 0.896 | 0.11 | 0.353 | 0.01 | 0.932 |
| NPI sum | -0.08 | 0.497 | -0.06 | 0.619 | -0.09 | 0.438 | 0.03 | 0.836 | -0.04 | 0.719 | -0.08 | 0.529 | -0.08 | 0.512 |
| **CSF total proteins** | 0.16 | 0.17 | **-0.52** | **3.0·10^-6^** | -0.22 | 0.067 | **-0.45** | **9.6·10^-5^** | **-0.55** | **5.2·10^-7^** | 0.18 | 0.14 | -0.23 | 0.057 |

**Table S1 Spearman’s correlations between αS-SAA kinetic parameters and neuropsychological scores at baseline.** Correlation coefficients having an unadjusted p-value below 0.05 are highlighted in bold. MMSE: Mini-Mental State Examination; HDS-IT: HIV-dementia scale, Italian version; CDT: Clock Drawing Test; TMT-A: Trail Making Test A; TMT-B: Trail Making Test B; DigitspanFORW: digit span forward; DigitspanBACK: digit span backward; RAVLT: Rey Auditory Verbal Learning Test; RAVLTimm: Rey Auditory Verbal Learning Test immediate recall; RAVLTdel: Rey Auditory Verbal Learning Test delayed recall; RAVLT-TrueRecog: Rey Auditory Verbal Learning Test true recognition; RAVLT-FalseRecog: Rey Auditory Verbal Learning Test false recognition; SR: Short story recall of Anna Pesenti; Draw copy: copy of drawings without landmarks from the Mental Deterioration Battery; Draw copy land: copy of drawings with landmarks from the Mental Deterioration Battery; Letter fluency: phonemic fluency; Cat fluency: category fluency; CDR: Clinical Dementia Rating scale; NPI: Neuropsychiatric Inventory; NPI sum = sum of the items of the NPI. TTT: time-to-threshold (time to reach 3,000 RFU); Fmax: maximum fluorescence; F24h: fluorescence at 24 h; AUFC: area under the fluorescence curve; Smax: maximum slope of the fluorescence curve; TSmax: time to reach the maximum slope; AUFCdydx: area under the derivative of the fluorescence curve.

|  | **αS-SAA kinetic parameters** | | | | | | | | | | | | | |
| --- | --- | --- | --- | --- | --- | --- | --- | --- | --- | --- | --- | --- | --- | --- |
| **Neuropsychological  tests** | **TTT** | | **Fmax** | | **F24h** | | **AUFC** | | **Smax** | | **TSmax** | | **AUFCdydx** | |
|  | ρ part. | p-value | ρ part. | p-value | ρ part. | p-value | ρ part. | p-value | ρ part. | p-value | ρ part. | p-value | ρ part. | p-value |
| MMSE | -0.09 | 0.453 | -0.07 | 0.560 | -0.05 | 0.667 | -0.03 | 0.824 | -0.08 | 0.485 | -0.09 | 0.483 | -0.07 | 0.568 |
| HDS-IT | -0.12 | 0.369 | -0.23 | 0.080 | -0.23 | 0.072 | -0.10 | 0.431 | -0.21 | 0.106 | -0.12 | 0.368 | **-0.27** | **0.035** |
| CDT | -0.02 | 0.888 | 0.01 | 0.957 | 0.04 | 0.743 | 0.09 | 0.467 | -0.03 | 0.796 | -0.02 | 0.890 | 0.06 | 0.616 |
| TMT-A | 0.08 | 0.649 | -0.06 | 0.715 | -0.11 | 0.535 | -0.02 | 0.901 | -0.09 | 0.586 | 0.07 | 0.670 | -0.08 | 0.642 |
| TMT-B | -0.09 | 0.604 | -0.24 | 0.176 | -0.27 | 0.122 | 0.03 | 0.869 | -0.29 | 0.095 | -0.08 | 0.639 | -0.24 | 0.165 |
| DigitspanFORW | -0.14 | 0.367 | 0.04 | 0.786 | -0.06 | 0.714 | 0.10 | 0.533 | 0.08 | 0.624 | -0.14 | 0.369 | -0.08 | 0.608 |
| DigitspanBACK | **-0.38** | **0.011** | 0.02 | 0.902 | -0.16 | 0.296 | 0.22 | 0.151 | 0.05 | 0.728 | **-0.38** | **0.011** | -0.18 | 0.236 |
| RAVLT imm | 0.04 | 0.786 | -0.01 | 0.972 | 0.02 | 0.904 | -0.07 | 0.637 | 0.00 | 0.976 | 0.04 | 0.789 | 0.01 | 0.931 |
| RAVLT del | 0.04 | 0.791 | -0.09 | 0.549 | 0.08 | 0.584 | -0.10 | 0.527 | -0.09 | 0.543 | 0.04 | 0.794 | 0.09 | 0.563 |
| RAVLT-TrueRecog | -0.04 | 0.814 | 0.08 | 0.588 | 0.18 | 0.246 | 0.08 | 0.613 | 0.10 | 0.538 | -0.05 | 0.761 | 0.18 | 0.240 |
| RAVLT-FalseRecog | -0.05 | 0.731 | 0.06 | 0.683 | -0.01 | 0.955 | 0.15 | 0.341 | 0.04 | 0.809 | -0.03 | 0.833 | 0.02 | 0.896 |
| SR | -0.07 | 0.700 | 0.06 | 0.746 | 0.09 | 0.639 | 0.05 | 0.789 | 0.03 | 0.853 | -0.07 | 0.715 | 0.08 | 0.667 |
| Draw copy | -0.10 | 0.502 | 0.10 | 0.509 | -0.05 | 0.762 | 0.09 | 0.545 | 0.09 | 0.528 | -0.10 | 0.522 | -0.04 | 0.783 |
| Draw copy land | -0.15 | 0.313 | 0.08 | 0.590 | 0.00 | 0.996 | 0.13 | 0.392 | 0.13 | 0.386 | -0.15 | 0.319 | 0.00 | 0.999 |
| Letter fluency | -0.03 | 0.864 | 0.04 | 0.782 | 0.01 | 0.931 | 0.00 | 1.000 | 0.09 | 0.539 | -0.02 | 0.877 | 0.00 | 0.990 |
| Cat fluency | -0.12 | 0.418 | 0.08 | 0.591 | 0.02 | 0.906 | 0.07 | 0.616 | 0.07 | 0.659 | -0.12 | 0.426 | 0.02 | 0.888 |
| CDR | 0.10 | 0.438 | 0.22 | 0.087 | 0.20 | 0.124 | 0.11 | 0.399 | 0.20 | 0.119 | 0.10 | 0.466 | 0.24 | 0.060 |
| **NPI domains** |  |  |  |  |  |  |  |  |  |  |  |  |  |  |
| Anxiety | -0.07 | 0.588 | 0.11 | 0.373 | -0.01 | 0.913 | 0.17 | 0.180 | 0.13 | 0.294 | -0.06 | 0.634 | -0.01 | 0.961 |
| Delusions | **-0.34** | **0.006** | -0.10 | 0.435 | -0.17 | 0.163 | 0.20 | 0.108 | -0.08 | 0.495 | **-0.33** | **0.007** | -0.16 | 0.184 |
| Hallucinations | **-0.28** | **0.021** | 0.01 | 0.929 | -0.05 | 0.673 | **0.25** | **0.037** | 0.03 | 0.792 | **-0.30** | **0.014** | -0.06 | 0.643 |
| Depression/dysphoria | 0.13 | 0.288 | 0.02 | 0.854 | 0.04 | 0.745 | -0.11 | 0.373 | 0.03 | 0.838 | 0.14 | 0.252 | 0.03 | 0.783 |
| Agitation/aggression | -0.05 | 0.692 | -0.13 | 0.307 | -0.14 | 0.258 | -0.08 | 0.546 | -0.10 | 0.401 | -0.04 | 0.734 | -0.12 | 0.333 |
| Euphoria | -0.22 | 0.073 | -0.14 | 0.255 | -0.19 | 0.122 | 0.04 | 0.740 | -0.13 | 0.289 | -0.22 | 0.070 | -0.20 | 0.113 |
| Apathy/indifference | 0.00 | 0.988 | 0.06 | 0.603 | 0.03 | 0.829 | 0.08 | 0.543 | 0.11 | 0.364 | -0.01 | 0.955 | 0.05 | 0.708 |
| Disinhibition | -0.13 | 0.277 | 0.16 | 0.197 | 0.03 | 0.831 | 0.23 | 0.065 | 0.15 | 0.221 | -0.13 | 0.278 | 0.05 | 0.675 |
| Irritability | 0.10 | 0.434 | **0.26** | **0.032** | 0.24 | 0.049 | 0.04 | 0.738 | **0.28** | **0.021** | 0.09 | 0.481 | 0.23 | 0.063 |
| Aberrant motor behavior | -0.06 | 0.637 | 0.06 | 0.633 | -0.07 | 0.585 | 0.04 | 0.744 | 0.05 | 0.659 | -0.07 | 0.596 | -0.05 | 0.691 |
| Nighttime behaviors | **-0.31** | **0.010** | 0.03 | 0.782 | -0.02 | 0.848 | **0.27** | **0.027** | 0.05 | 0.715 | **-0.31** | **0.011** | -0.01 | 0.946 |
| Appetite/eating | 0.09 | 0.453 | 0.08 | 0.529 | 0.02 | 0.886 | 0.01 | 0.947 | 0.07 | 0.572 | 0.09 | 0.463 | 0.04 | 0.737 |
| NPI sum | -0.14 | 0.243 | 0.1 | 0.406 | -0.04 | 0.772 | 0.18 | 0.139 | 0.14 | 0.259 | -0.14 | 0.249 | -0.02 | 0.888 |

**Table S2. Spearman’s partial correlations between αS-SAA kinetic parameters and neuropsychological scores at baseline adjusted for CSF total protein concentration.** Correlation coefficients having an unadjusted p-value below 0.05 are highlighted in bold. MMSE: Mini-Mental State Examination; HDS-IT: HIV-dementia scale, Italian version; CDT: Clock Drawing Test; TMT-A: Trail Making Test A; TMT-B: Trail Making Test B; DigitspanFORW: digit span forward; DigitspanBACK: digit span backward; RAVLT: Rey Auditory Verbal Learning Test; RAVLT imm: Rey Auditory Verbal Learning Test immediate recall; RAVLT del: Rey Auditory Verbal Learning Test delayed recall; RAVLT-TrueRecog: Rey Auditory Verbal Learning Test true recognition; RAVLT-FalseRecog: Rey Auditory Verbal Learning Test false recognition; SR: Short story recall of Anna Pesenti; Draw copy: copy of drawings without landmarks from the Mental Deterioration Battery; Draw copy land: copy of drawings with landmarks from the Mental Deterioration Battery; Letter fluency: phonemic fluency; Cat fluency: category fluency; CDR: Clinical Dementia Rating scale; NPI: Neuropsychiatric Inventory; NPI sum = sum of the items of the NPI. TTT: time-to-threshold (time to reach 3,000 RFU); Fmax: maximum fluorescence; F24h: fluorescence at 24 h; AUFC: area under the fluorescence curve; Smax: maximum slope of the fluorescence curve; TSmax: time to reach the maximum slope; AUFCdydx: area under the derivative of the fluorescence curve.
